# Polychlorinated Biphenyls and Semen Quality in Healthy Young Men Living in a Contaminated Area

**DOI:** 10.3390/toxics12010006

**Published:** 2023-12-20

**Authors:** Francesco Donato, Matteo Rota, Elisabetta Ceretti, Gaia Claudia Viviana Viola, Monica Marullo, Danilo Zani, Angela Amoresano, Carolina Fontanarosa, Michele Spinelli, Stefano Lorenzetti, Luigi Montano

**Affiliations:** 1 Unit of Hygiene, Epidemiology and Public Health, Department of Medical and Surgical Specialties, Radiological Sciences and Public Health, University of Brescia, 25123 Brescia, Italy; francesco.donato@unibs.it (F.D.); gaia.viola@unibs.it (G.C.V.V.); monica.marullo@unibs.it (M.M.); 2 Unit of Biostatistics and Bioinformatics, Department of Molecular and Translational Medicine, University of Brescia, 25123 Brescia, Italy; matteo.rota@unibs.it; 3 Unit of Urology, Department of Medical and Surgical Specialties, Radiological Sciences and Public Health, University of Brescia, 25123 Brescia, Italy; danilo.zani@unibs.it; 4 Department of Chemical Sciences, University of Naples Federico II, 80126 Naples, Italy; angela.amoresano@unina.it (A.A.); carolina.fontanarosa@unina.it (C.F.); michele.spinelli@unina.it (M.S.); 5 Department of Food Safety, Nutrition and Veterinary Public Health, Italian National Institute of Health (ISS), 00161 Rome, Italy; stefano.lorenzetti@iss.it; 6 Andrology Unit and Service of Lifestyle Medicine in UroAndrology, Local Health Authority (ASL) Salerno, Coordination Unit of the Network for Environmental and Reproductive Health (EcoFoodFertility Project), “Oliveto Citra Hospital”, 84020 Salerno, Italy; 7 PhD Program in Evolutionary Biology and Ecology, University of Rome Tor Vergata, 00133 Rome, Italy

**Keywords:** polychlorinated biphenyls, persistent organic pollutants, semen quality, sperm motility, sperm normal morphology, sperm concentration

## Abstract

Polychlorinated biphenyls (PCBs) are persistent organic pollutants and endocrine disruptors that have been implicated in potential damage to human semen. However, the studies conducted so far provide contrasting results. Our study aimed to investigate the associations between PCB serum and semen levels and semen quality in high school and university students living in a highly PCB-polluted area of Italy. Subjects with a normal body mass index who did not make daily use of tobacco, alcohol, drugs, or medication were selected. All participants provided a fasting blood and a semen sample. Gas chromatography-mass spectrometry was used to determine the concentrations of 26 PCB congeners. The concentrations of PCB functional groups and total PCBs were also computed. A total of 143 subjects (median age 20, range 18–22 years) were enrolled. The median total PCB concentrations were 3.85 ng/mL (range 3.43–4.56 ng/mL) and 0.29 ng/mL (range 0.26–0.32 ng/mL) in serum and semen, respectively. The analysis of the associations between sperm PCB concentration and semen parameters showed (a) negative associations between some PCB congeners, functional groups and total PCBs and sperm total motility; (b) negative associations of total PCBs with sperm normal morphology; and (c) no association of PCBs with sperm concentration. Subjects at the highest quartile of semen total PCB concentration had 19% and 23% mean reductions in total motility and normal morphology, respectively, compared to those at the lowest quartile. The analysis of the associations of serum PCB levels with sperm parameters yielded null or mixed (some positive, other negative) results. In conclusion, the present study provides evidence of a negative effect of some PCB congeners and total PCBs in semen on sperm motility and normal morphology. However, the associations between the concentration of serum and semen PCB congeners and functional groups and sperm quality parameters were inconsistent.

## 1. Introduction

Polychlorinated biphenyls (PCBs) are a group of 209 compounds (congeners) classified as persistent organic pollutants (POPs) under the Stockholm Convention in 2001 [1]. They have been produced in past century up to the 1970s–1980s, when they were banned in most countries due to their toxicity and environmental persistence [1]. Despite their banning in most countries, environmental and occupational exposure to these pollutants, mainly deriving from building materials, contaminated foods, particularly seafoods, and electrical equipment, is still a matter of concern [2,3,4,5]. Indeed, high levels of these compounds have been found in people who live or work in areas where electronic wastes are collected and managed [6,7,8,9].

Several studies on humans have shown an association between PCB exposure and metabolic, endocrine, immunological, neurological, and cardiovascular diseases [10,11,12,13,14,15]. Furthermore, PCBs have been classified by the International Agency for Research on Cancer as carcinogenic to humans [16].

An increase in male infertility has been documented in last decades, although its causes are still debated [17]. Environmental compounds, particularly endocrine-disrupting chemicals (EDCs), including PCBs, are considered as the major determinants of low sperm quality in humans by many authors [17]. Many experimental animal studies and in vitro tests have shown deleterious effects of PCBs on cells and various organs and systems, including reproductive and developmental effects, as summarized by the WHO [18]. Accordingly, observational human studies found that PCB levels in serum and/or seminal plasma were associated with semen quality parameters, particularly sperm count, normal morphology, and motility [6,19].

A chemical factory (Caffaro) located in Brescia, North Italy produced organochlorine compounds, including PCBs, from the 1930s to 1984. PCBs were discharged into irrigation channels and thence accumulated in the soil of an agricultural area and entered the food chain. First studies carried out in 2001–2003 showed high PCB contamination in soil, surface water, animals, vegetables, and humans, mainly in people who had consumed locally produced animal food [20,21]. Particularly, the total PCB concentrations were 10–8300 (median 500) μg/kg in the soil of the most polluted area of Brescia and 64–23,819 (median 566) ng/g of lipid in animal food produced in the same area [21]. Lower PCB values were found in plant food and surface waters and no contamination in drinkable water.

Despite the subsequent public health interventions for preventing environmental and human PCB contamination, high serum levels of various PCB congeners and total PCBs were still found in people living in the city in a 2013-2014 survey [22]. Increased risks of cardiovascular and neurological diseases, but not of endocrine or metabolic disorders, were found in people living in the area with high PCB serum levels [23,24,25,26]. However, no studies have been performed on semen parameters and PCB exposure in this area so far. 

This study aimed to quantify PCB serum and semen levels and investigate the relationship between serum and sperm concentration of various PCB congeners, functional groups, and total PCBs with sperm quality parameters in healthy young men living in a highly industrialized and PCB-polluted area in North Italy.

## 2. Materials and Methods

### 2.1. Study Population

This FASt (“Fertilità, Ambiente, alimentazione, STile di vita”) study was a randomized controlled trial (registered on ClinicalTrials.gov Protocol Registration and Results System; receipt release date: 15 February 2019; n. J59D1600132001) that aimed to evaluate the effects of a dietary and physical activity intervention on the semen quality of healthy young men living in some highly polluted areas of Italy, including Brescia [27]. The RCT was conducted between April 2018 and June 2019 and the study design was detailed elsewhere [27]. Briefly, a sample of high school and university male students aged 18–22 years were enrolled. According to the aim of the study, which was to assess the relationship between environmental risk factors and semen quality in healthy adolescents living in a polluted area, we used severe restriction rules for participating subjects to reduce the risk of confounding bias. Therefore, we excluded individuals with major risk factors for semen quality, such as history of chronic diseases, regular (daily) use of tobacco, alcohol, drug or medicine and overweight/obesity. 

All the participants were interviewed on their medical history and provided a blood sample for the analysis of some common biochemical parameters: fast glycaemia, protein levels, blood cell counts and characterization, etc. Only those without diagnosis or suspicion of chronic disease were included in the study. 

Upon their recruitment, all subjects underwent measurement of weight, height, and abdominal circumference and filled in three standardized questionnaires: the European Prospective Investigation into Cancer and Nutrition (EPIC) questionnaire, a semiquantitative food-frequency questionnaire for evaluating the usual intake of macro- and micro-nutrients [28], the “PREvención con DIeta MEDiterránea” (PREDIMED) questionnaire for evaluating adherence to the Mediterranean diet [29], and the International Physical Activity Questionnaire (IPAQ) for evaluating physical activity [30]. Both the PREDIMED and IPAQ scores were considered as potential confounders of the associations between PCB serum and semen levels and semen quality. Conversely, we did not consider here the macro- and micro-nutrients intake assessed through the EPIC questionnaire, as we did not find any association between them and semen parameters [31]. Due to the high PCB environmental pollution in the Brescia province in the past, we restricted the present analysis to the FASt study participants enrolled in the Brescia area.

### 2.2. Biological Sampling and Storage

The fasting blood specimens were collected by trained healthcare staff, centrifuged within 5 h of collection and the serum separated. We prepared small serum aliquots (0.5 mL) in vials and stored them at −80 °C until analysis for the evaluation of biochemistry parameters and the quantification of PCB congeners.

Each semen sample was collected in a sterile container through masturbation, after at least 3 days and at most 5 days of abstinence from sexual activity. The semen sample was delivered to the laboratory within 30–40 min after collection, whereas a portion of semen (<50 μL) was processed immediately for the spermiogram. 

All the analyses were performed by an expert urologist under the same conditions, according to WHO Manual 2010, using a classical microscope for optical evaluation with a Makler counting chamber and two automated semen analysers: SQA-V GOLD (Medical Electronic Systems Ltd. Israel) following the manufacturer’s guidelines and the Lenshooke Semen X1 Pro system (Bonraybio Co., LTD. Dali Dist., Taichung City, Taiwan), an innovative automatic analytical certified CE IVD system, equipped with a microscopic integrated optics and artificial intelligence system. The following parameters were measured: sample volume, sperm concentration, total and progressive motility, and proportion of morphologically normal spermatozoa (% cells with normal morphology).

The serum and semen samples were taken at enrolment (baseline) and after 4 months, according to the RCT design: since spermatogenesis takes about 64 days, the RCT times were established to evaluate the effect of the 4-month intervention on sperm parameters at the end of the intervention (4th month) [27]. 

The PCB and semen parameters analyses were performed on the samples taken at baseline and after 4 months for two reasons: (a) to assess the agreement between the two measures on an individual basis (internal validity of the study), and (b) to increase the precision of the estimates and the statistical power of the tests regarding the relationships between PCB concentrations in serum and semen and quality parameters.

### 2.3. PCB Analysis

The PCBs were measured in the serum and semen via mass spectrometry techniques at the Department of Chemical Sciences, University of Naples Federico II, Italy. 

The reagents and standards, acetonitrile, hexane, and cyclohexane were HPLC-grade. The water was purified using a Milli-Q system. The PCB congeners (IUPAC numbers 18, 28, 31, 77, 81, 95, 99, 101, 105, 110, 114, 118, 123, 126, 128, 138, 146, 149, 151, 153, 156, 157, 167, 169, 170 and 180 in isooctane at 100 µg/mL) were purchased from AccuStandard (New Haven, CT, USA). The acetonitrile (ACN) was purchased from Romil (Waterbeach, Cambridge, UK) and the formic acid (HCOOH) from Millinckrodt Baker (Phillipsburg, NJ, USA).

The stock standard solution of the PCB congeners was prepared in cyclohexane at a concentration of 1 µg/mL. The sample aliquots (200 µL) were added with 10 mL of purified water to the centrifuge tube. After letting stand for 30 min, 10 mL of acetonitrile was added and vortexed for 3 min. The samples were centrifuged for 5 min at 5000 rpm. The supernatants were dried under nitrogen, and the residue was dissolved in 1 mL of cyclohexane, then filtered through a PTEE filter (0.22 µm). The supernatant was evaporated to dryness under a stream of nitrogen in a 40 °C water bath. Afterwards, 0.5 g of NaCl was added and shaken vigorously for several seconds to provide good phase separation. The tubes were centrifuged for 5 min at 5000 rpm and the supernatants directly used for GC-MS/MS analyses. 

An Agilent 7693 autosampler was coupled to a QQQ system Agilent 7890A. The column was an Agilent HP-5 MS UI (30 m × 0.25 mm, 0.25 μm) (p/n 19091S-433 UI). The oven temperature was 60 °C held for 1 min, then increasing at 40 °C/min to 120 °C, then increasing at 5 °C/min to 275 °C. The carrier gas was helium at a flow rate of 1.0 mL/min. The injection port temperature was 280 °C and the injection volume was 1.0 μL in splitless injection mode. The MS conditions of the Agilent 7000C Triple Quadrupole GC/MS System were as follows: ion source EI ionization voltage 70 eV, ion source temperature 280 °C, quadrupole temperature Q1 150 °C Q2 150 °C, interface temperature 280 °C, solvent delay 10.0 min. The following software was used: Agilent MassHunter Data Acquisition Software (Ver. B.04.00) and MassHunter Workstation Software for Qualitative Analysis (Ver. B.03.01).

Two transitions were determined by GC-MS/MS for each compound and the collision energy was optimized for each. 

The quantitative analyses were performed using external standards. Six standard mixtures at known concentration were prepared for each class of compound and analysed via GC-MS/MS to build up calibration curves. Three replicates were recorded for each point with coefficient variations lower than 10%. The values of concentrations were obtained by interpolating the recorded areas on the related calibration curves. For all pollutants, the limit of detection for each analyte was 0.001 ng/mL, while the limit of quantification was 0.003 ng/mL for each congener in both serum and semen. A minimum concentration equal to half the quantification limit (0.0005 ng/mL) was assigned for congeners detected in at least 30% of the subjects.

The functional PCB groups were classified according to Cocco et al. [32] as follows: congeners with immunotoxic activity (PCBs 138, 153 and 180); low-chlorinated PCBs with pseudo-oestrogen activity (PCBs 28, 52 and 153); highly chlorinated PCBs with anti-oestrogenic activity (PCBs 170, 180 and 194); and PCBs that can induce phenobarbital (PCBs 101, 153, 180 and 194). Furthermore, since 12 PCB congeners have activity similar to that of polychlorinated dibenzo-*p*-dioxins due to the share of a common mechanism of action via the aryl hydrocarbon receptor (AhR), we also computed the total dioxin-like PCBs as the sum of PCBs 77, 81, 105, 114, 118, 123, 126, 156, 157, 167, 169, and 189. We also computed the ratio of pseudo-oestrogenic to anti-oestrogenic activity PCBs, due to their potential opposite endocrine effects on spermatogenesis. The total PCB serum concentration was computed as the sum of PCB congeners measured.

## 3. Statistical Analysis

Study participants’ characteristics and semen parameters were described through median and interquartile range (IQR), while serum and semen levels of PCB congeners detected in at least 30% of the subjects, functional PCB groups and total PCBs concentrations were summarised through median and range.

Since the distribution of each PCB congener, as well as functional PCB groups and total PCBs concentrations, was skewed and non-normal, the Spearman rank correlation coefficient was used to assess the correlation between serum and semen PCBs exposure, and between PCBs exposure and semen parameters.

All the semen and blood samples taken at the enrolment (baseline) and after 4 months were analysed. A linear mixed model with robust variance estimation was fitted to assess the relationships between PCB congeners, functional PCB groups and total PCB levels, and sperm concentration, while a generalized linear mixed model (GLMM) with the Poisson family was used to evaluate cell motility and morphologically normal spermatozoa counts [33], with overall cell number as the offset. The total PCB exposure was further categorized into quartiles to show the contrasts between low and high exposure. 

A positive false-discovery rate (pFDR) correction [34] was applied to account for test multiplicity, in other words the potential inflation of the type I error arising from the test of all the considered PCB congeners, functional groups, and total PCB exposure. The pFDR correction allowed for the computation of adjusted *p*-values, the so-called *q*-values, representing the expected pFDR obtained by rejecting the null hypothesis for any result with an equal or smaller *q*-value.

To ensure comparability across different PCB congeners, the standardized regression coefficients were computed. The linear models provided coefficients as measures of association, with positive and negative coefficients indicative of improvement and decline in sperm concentration, respectively, whereas the Poisson models provided incidence rate ratios (IRRs) as measures of associations, with values greater or lower than 1 indicative of improvement and decline in sperm motility or morphologically normal cells, respectively. All the models were adjusted for seasonality since semen parameters declined in winter, compared to the other seasons.

A *p*-value < 0.05 was deemed to be statistically significant. All the analyses were carried out with R version 4.2.1.

## 4. Results

A total of 143 healthy young men living in Brescia province, North Italy, had a complete physical and semen quality evaluation and provided valid questionnaires. Their characteristics are summarized in Table 1. At baseline (enrolment) the subjects had a median age of 20 years, a median BMI of 22.2, a relatively low PREDIMED score and a relatively high IPAQ score, mainly due to regular sports practice.

The serum and semen measures at baseline and the fourth month of PCB congeners, functional groups, and total PCBs are summarized in Table 2. Some serum and/or semen data were missing at each observation time, due to subject loss or insufficient quantity of semen or serum for PCB analyses. The semen PCB concentrations were found to be lower than those in serum for all the congeners, with more than half of them being undetectable in semen, especially the highly chlorinated ones. Among the functional groups, the highest concentration was found for the dioxin-like PCBs, in both serum and semen. At baseline, the median total PCB concentration was 3.85 ng/mL (range 3.43–4.56 ng/mL) and 0.29 ng/mL (range 0.26–0.32 ng/mL) in the serum and semen, respectively.

Null or weak correlations were found between baseline serum and semen levels of PCB congeners, functional groups and total PCBs (Supplementary Table S1). Some of the Spearman correlation coefficients were positive and others negative, with a weak correlation emerging for total PCBs (r = −0.13). 

The distributions of sperm concentration, total motility, and proportion of morphologically normal spermatozoa by levels of PCB functional groups and of total PCBs in serum are graphically represented trough scatter diagrams (Supplementary Figures S1–S3). Wide dispersions emerged for all the outcomes, with low to moderate Spearman correlation coefficients (r < 0.4 for all measures).

The results of the mixed models assessing the relationships between serum PCB congeners, functional groups, and total PCBs as predictors and sperm concentration, total motility, and normal morphology as outcomes are shown in Table 3. Considering only the non-null estimates and the results with statistically significant *q*-values, we observed: (a) for sperm concentration, no associations; (b) for sperm motility, positive (motility improvement) associations for PCBs 81, 95, 105, 114, 118, 180, highly-chlorinated anti-oestrogenic and dioxin-like PCBs, and negative (motility decline) associations for PCBs 18, 99, 138, 146, 149 and the highest compared to the lowest quartile of total PCBs; (c) for sperm normal morphology, positive (improvement of morphologically normal cell proportion) associations for PCBs 126, 151, 170, highly-chlorinated anti-estrogenic and dioxin-like PCBs, and a negative association (morphologically normal spermatozoa decline) for PCB 149.

Table 4 shows the results on sperm PCB congeners. Considering only the non-null estimates and the results with statistically significant *q*-values, we observed: (a) for sperm concentration, no associations; (b) for sperm motility, positive (motility improvement) associations for PCBs 28 and 157, and negative associations (motility decline) for PCBs 31, 114, the immunotoxic, phenobarbital-inducer PCBs, and total PCBs; (c) for sperm normal morphology, a positive (improvement in morphologically normal cell proportion) association for PCBs 99 and 157, and a negative association (normal morphology decline) for PCBs 31, 81, 114, 156, the dioxin-like and the total PCBs. The analysis of total PCB quartiles shows that subjects with the highest semen PCB concentration had a mean reduction of 19% in total motility (from 12% to 25%) and a 30% decrease in normal morphology proportion (from 27% to 42%) when compared to those in the lowest quartile.

The analysis of the associations between serum and semen PCB concentrations and sperm progressive motility produced similar results as that of sperm total motility (Supplementary Table S2).

Similar results emerged in sensitivity analyses performed including all the subjects’ characteristics reported in Table 1 as possible confounders in the regression models.

## 5. Discussion

This study shows various associations, some positive and others negative, between serum and semen PCB congeners, functional groups, and sperm quality parameters, particularly total motility.

Despite the discontinuation of PCB production in most countries 40–50 years ago, PCBs are still of public health concern. This is primarily due to the long persistence of PCBs produced in the past and of those released as by-products of certain industrial processes, including the manufacture of paints and dyes, in recent years. Animal-based foods, particularly marine foods, but also meat and dairy products, have been traditionally considered the main source of PCB contamination in humans. However, PCB concentrations in foods have substantially declined in the past 20 years. Consequently, recent assessments indicate that PCB dietary exposures are now comparable with PCB inhalation exposures [35].

The potential adverse impact of PCBs on sperm quality and consequently human fertility has been hypothesised, mainly attributed to their endocrine-disrupting activity. Consequently, PCBs have been classified as EDCs, i.e., “exogenous chemicals, or mixtures of chemicals, that can interfere with any aspect of hormone action”, according to the Endocrine Society [36]. Although experimental animal studies sustained this hypothesis, the results of epidemiological studies in humans are inconclusive [19,37]. For instance, a large, multicentre, Inuit and European study provided no evidence of hormone-like activity in PCB 153, which is the most investigated PCB congener in humans; its serum concentration was not consistently related to either endogenous or exogenous hormone activity [38]. 

In addition to their endocrine activity, PCBs might have other negative effects on sperm quality. These chemicals consist of 209 diverse congeners, with different toxicity and biological activity, according to their degree of chlorination and spatial structure [39]. Indeed, mixtures of 50 PCB congeners and more (Aroclors) were produced in the past or discharged as by-products of industrial processes. Among them, different functional groups have been identified, according to their activity, from immune system interference to dioxin-like activity via the AhR, with possible activation of various enzymes and metabolic ways [32]. For this reason, we focused our analysis on both single PCB congeners and functional groups, in addition to the sum of total PCBs.

We found mixed results on the associations between PCB congeners and sperm quality parameters, from negative to positive or inconclusive associations. These findings were not unexpected, as previous published studies found similar discrepancies between PCB congeners. Of the 23 epidemiological studies reviewed by Ermler and Kortenkamp [19], 9 reported null findings, 4 reported mixed findings, with decline in semen quality for some congeners and improvement for others, and 3 reported improvement and 8 reported declines in one or more semen parameters in relation to various PCBs. Although some PCB congeners might theoretically have a “true” positive effect on sperm quality, for instance for their anti-oestrogenic activity, these discrepancies are more likely due to differences in study design, people enrolled, choice of PCB congeners, lack of control for confounding, and other related factors. Particularly, the number of investigated PCB congeners ranged from 1 (usually PCB 153) to 57 congeners, and it is plausible that the larger the number of PCB congeners analysed, the higher the probability of achieving mixed results (positive results for some and negative for other congeners) by chance only. To minimize the potential for erroneously rejecting the null hypothesis of a lack of association, or effect, we employed the false discovery rate correction for multiple testing. While this approach can aid in reducing the likelihood of chance-related associations, it does not entirely eliminate the risk. Therefore, we cannot exclude that certain observed “positive” or “negative” effects of PCB congeners in our study may be attributed to chance, thus requiring confirmation.

This study aimed to investigate both serum and sperm PCB concentrations, functional groups, and total PCBs. Our findings revealed null or low correlations between PCB concentrations in serum and semen, in agreement with others [6], suggesting that the diffusion of PCBs is not uniform in human organs and tissues. Although the majority of PCBs exhibited lower concentrations in sperm compared to serum (with some compounds detectable in serum but not in sperm), we found statistically significant negative associations between the concentration of certain PCB congeners in sperm and semen motility and normal morphology. However, in contrast to the biological hypothesis of a toxic effect of PCBs on human sperm, we found positive associations between serum concentrations of various PCB congeners (namely, PCBs 81, 95, 105, 114, 126, 118, 151, 170, 180), highly chlorinated PCBs with anti-oestrogenic activity, and dioxin-like PCBs with sperm motility and/or sperm normal morphology.

Anyway, two critical points should be considered in determining the potential for a cause–effect relationship between PCB exposure and sperm quality. First, there are inconsistent results of the studies on the association between single PCB congeners and sperm parameters. In a systematic review conducted by Ermler and Kortenkamp, the evidence of adverse effects of PCBs on sperm parameters in humans was deemed “robust” for PCBs 118 and 169, “moderate” for PCBs 126, 132, 149, and 153, “slight” for PCB 77, and “indeterminate” for PCB 180 [19]. The findings of our study diverge from the conclusions drawn by Ermler and Kortenkamp in their review. Our study found a “positive” association of PCB 118 and 126 and no associations between PCBs 169 and 153, as measured in both serum and sperm and the parameters of semen quality. 

Other studies reported contrasting results, with negative associations between sperm parameters and PCB for other congeners. A recent study conducted among 20–50-years-old men living near an electronic waste site in China reported negative associations between semen levels of PCBs 44, 66, 105, and 153 and sperm concentration, as well as between serum levels of PCB 153 and sperm progressive motility. However, the study did not provide any data on the other sperm quality parameters, probably because they exhibited null associations [6]. 

A second critical point regarding the potential cause–effect relationship between PCB exposure and semen quality is the limited consistency observed in the association between semen parameters and PCB concentrations in serum or semen. In Ermler and Kortenkamp’s review, the findings revealed a lack of agreement among the results of the studies on the effect of PCB exposure on sperm quality parameters: (a) for sperm concentration, three studies reported a decline, three studies indicated an improvement, and fifteen studies found no association; (b) for sperm motility, nine studies reported a decline, one study observed an improvement, and twelve studies found no association; (c) for sperm normal morphology, two studies reported a decline, one study indicated an improvement and twelve studies found no association [19]. According to these results, sperm motility seems to be the most sensitive semen parameter in relation to potential PCB adverse effects, although the majority of studies found no effect. However, it is noteworthy that all but one of the studies solely evaluated PCB blood measurements, whereas it is theoretically plausible that the concentration of these chemicals directly in semen may be a more appropriate biomarker for evaluating the potential adverse effects of PCBs on semen quality. 

The relationship between PCB exposure and fertility in humans is uncertain. Reproductive abnormalities were found in populations exposed to high concentrations of PCBs or other POPs, including reduced semen quality and testicular cancer in males, menstrual cycle abnormalities and spontaneous abortions in females, prolonged waiting time to pregnancy, reduced birth weight of offspring, skewed sex ratio, and altered age of sexual development. However, it should be noted that these reproductive alterations have been minor, if any, in the general population exposed to present, lower levels of these chemicals [40]. A study conducted on Inuit and European populations showed no association between PCB serum levels and delayed conception, which is indicative of low fertility, in three out of four groups investigated [38]. 

This study has various strengths. First, the study participants were not selected on fertility as this was a population-based study. Second, only healthy young men without known risk factors for semen quality, such as regular use of tobacco, alcohol, drugs or medication, and obesity, were enrolled. In addition, a fixed time of abstinence before semen collection was required. Therefore, we are confident that no substantial confounding occurred in the study. Third, we analysed the concentrations of several PCB congeners in serum and semen in people living in an industrialised area in North Italy, where relatively high serum levels of PCBs and possibly of other POPs were observed. The analysis of PCB exposure and sperm quality parameters using biological samples (serum and semen) collected at the same time was not a drawback of the study because of the short time of spermatogenesis (about 3 months) and the long persistence of PCBs in the organism. Finally, the number of serum and semen samples analysed here is relatively high, and it is one of the largest among the studies carried out so far. 

The main limitation of this study, as well as of the other human studies, is that the PCB concentration in biological fluids was examined in an adult population. This may not be indicative of PCB exposure during the critical phases of foetal development and early life, which are considered to be the most vulnerable periods for fertility damage [41]. However, due to the long persistence of these chemicals in the human body, it is plausible that current levels of PCBs in serum and semen may also reflect maternal exposure during pregnancy and lactation, as suggested by others [42].

In summary, our study shows evidence of an adverse impact of specific PCB congeners, as well as total PCBs, in semen on sperm motility and morphology. However, contrasting results emerged on the associations between the concentrations of serum and semen PCB congeners and functional groups and sperm quality parameters.

## Figures and Tables

**Table 1:** Characteristics and semen parameters of 143 healthy young men living in Lombardy, North Italy.

	Baseline	4 Months
**Characteristics ^1^**		
Age (years)	20 (19, 21)	20 (19, 21)
IPAQ score (METs)	1960 (1055, 3182)	2547 (1112, 3808)
PREDIMED score	7 (5, 8)	8 (7, 10)
BMI (kg/m^2^)	22.2 (21.1, 24.2)	22.3 (21, 24.1)
Waist circumference (cm)	77 (74, 81)	77 (73, 80)
Abdominal circumference (cm)	84 (79, 87)	84 (79, 87)
**Semen parameters ^1^**		
Volume (mL)	3 (2, 3.35)	2.5 (1.8, 4)
Sperm concentration (10^6^/^mL^)	62 (27, 98)	64 (36, 91)
Total motility (%)	46 (28, 53)	36 (20, 48)
Progressive motility (%)	31 (14, 41)	26 (14, 37)
Cells with normal morphology (%)	6 (3, 10)	5 (3, 8.2)

^1^ Median (IQR). METs: Metabolic Equivalent of Tasks.

**Table 2:** Serum and semen levels of PCB congeners, functional PCB groups, and total PCBs: data are expressed in ng/mL as median (range).

PCB	Serum		Semen	
	Baseline	4 Months	Baseline	4 Months
18	0.062 (0.031–0.095)	0.031 (0.012–0.045)	0.027 (0.017–0.038)	0.041 (0.025–0.078)
28	0.095 (0.054–0.214)	0.050 (0.040–0.069)	0.032 (0.024–0.043)	0.030 (0.021–0.042)
31	0.052 (0.033–0.085)	0.031 (0.018–0.068)	0.015 (0.010–0.025)	0.006 (0.004–0.008)
77	0.046 (0.024–0.067)	0.017 (0.010–0.023)	Not detectable	Not detectable
81	0.045 (0.033–0.081)	0.014 (0.010–0.020)	0.0034 (0.0015–0.0050)	0.0005 (0.0005–0.0005)
95	0.025 (0.018–0.039)	0.022 (0.014–0.028)	0.012 (0.007–0.017)	0.011 (0.008–0.018)
99	0.029 (0.019–0.054)	0.025 (0.016–0.032)	0.023 (0.017–0.036)	0.014 (0.009–0.019)
101	0.039 (0.027–0.066)	0.024 (0.010–0.029)	Not detectable	Not detectable
105	0.40 (0.28–0.55)	0.47 (0.37–0.63)	Not detectable	Not detectable
110	0.39 (0.27–0.94)	0.47 (0.28–0.94)	Not detectable	Not detectable
114	0.060 (0.041–0.074)	0.101 (0.050–0.190)	0.022 (0.013–0.039)	0.021 (0.012–0.039)
118	0.056 (0.041–0.089)	0.060 (0.039–0.092)	0.019 (0.014–0.026)	0.001 (0.001–0.001)
123	0.034 (0.017–0.043)	0.050 (0.027–0.072)	0.018 (0.010–0.025)	0.016 (0.008–0.022)
126	0.102 (0.073–0.143)	0.045 (0.029–0.072)	Not detectable	Not detectable
128	0.54 (0.42–0.80)	0.49 (0.31–1.08)	Not detectable	Not detectable
138	0.039 (0.028–0.054)	0.043 (0.030–0.072)	Not detectable	Not detectable
146	0.11 (0.07–0.17)	0.08 (0.05–0.12)	Not detectable	Not detectable
149	0.051 (0.025–0.073)	0.051 (0.030–0.106)	Not detectable	Not detectable
151	0.15 (0.11–0.23)	0.19 (0.11–0.35)	0.035 (0.026–0.057)	0.027 (0.018–0.041)
153	0.12 (0.08–0.17)	0.17 (0.08–0.27)	0.022 (0.015–0.031)	0.018 (0.011–0.024)
156	0.105 (0.074–0.204)	0.134 (0.075–0.170)	0.036 (0.018–0.059)	0.038 (0.031–0.054)
157	0.080 (0.062–0.108)	0.072 (0.057–0.107)	0.0103 (0.0072–0.0133)	0.0077 (0.0065–0.0112)
167	0.065 (0.034–0.110)	0.067 (0.019–0.125)	Not detectable	Not detectable
169	0.56 (0.34–0.73)	0.63 (0.37–0.87)	Not detectable	Not detectable
170	0.49 (0.26–0.92)	0.54 (0.40–0.79)	Not detectable	Not detectable
180	0.041 (0.027–0.074)	0.050 (0.038–0.062)	Not detectable	Not detectable
Immunotoxic PCBs	0.20 (0.16–0.25)	0.26 (0.18–0.38)	0.023 (0.016–0.032)	0.019 (0.012–0.025)
Pseudo-oestrogen PCBs	0.21 (0.14–0.37)	0.2 (0.13–0.33)	0.055 (0.043–0.072)	0.048 (0.037–0.062)
Highly chlorinated anti-oestrogenic PCBs	0.54 (0.32–0.95)	0.59 (0.45–0.85)	Not detectable	Not detectable
Phenobarbital inducer PCBs	0.21 (0.15–0.27)	0.25 (0.14–0.34)	0.023 (0.016–0.032)	0.019 (0.012–0.025)
Dioxin-like PCBs	1.56 (1.36–1.86)	1.65 (1.42–2.01)	0.112 (0.097–0.136)	0.087 (0.075–0.108)
Pseudo-oestrogen to anti-oestrogenic PCB ratio	0.42 (0.16–0.70)	0.33 (0.24–0.66)	Not detectable	Not detectable
Total PCBs	3.85 (3.43–4.56)	4.09 (3.39–4.71)	0.29 (0.26–0.32)	0.24 (0.22–0.27)

**Table 3:** Results of season-adjusted mixed model of serum PCB congeners, functional PCB groups, and total PCBs on sperm concentration, total motility, and cells with normal morphology. Statistically significant detrimental effects of PCB congeners on sperm quality parameters are highlighted in red, favorable effects in green.

PCB	Sperm Concentration			Total Motility			Cells with Normal Morphology		
	$\hat{\beta }$ ^1^ (95% CI)	*p*-Value	*q*-Value ^2^	IRR ^1^ (95% CI)	*p*-Value	*q*-Value ^2^	IRR ^1^ (95% CI)	*p*-Value	*q*-Value ^2^
18	−13.0 (−23.0, −3.2)	0.01	0.09	0.88 (0.83, 0.94)	<0.001	0.001	0.93 (0.81, 1.06)	0.27	0.45
28	0.2 (−5.8, 6.2)	0.95	0.95	1.01 (0.96, 1.05)	0.83	0.98	0.91 (0.83, 1.00)	0.04	0.12
31	0.8 (−6.0, 7.6)	0.82	0.95	1.04 (0.98, 1.09)	0.19	0.30	1.02 (0.92, 1.13)	0.74	0.84
77	−4.7 (−14.0, 4.5)	0.32	0.55	1.00 (0.94, 1.06)	0.89	0.98	1.10 (0.97, 1.24)	0.13	0.27
81	−16.0 (−28.0, −3.8)	0.01	0.09	1.14 (1.06, 1.22)	<0.001	0.002	1.15 (0.98, 1.34)	0.08	0.21
95	2.1 (−2.8, 7.0)	0.40	0.62	1.01 (0.98, 1.05)	0.50	0.64	1.01 (0.94, 1.08)	0.81	0.86
99	−0.33 (−6.0, 5.4)	0.91	0.95	0.95 (0.91, 0.98)	0.01	0.02	0.97 (0.90, 1.04)	0.38	0.54
101	−4.9 (−12.0, 2.6)	0.20	0.49	1.03 (0.98, 1.08)	0.26	0.40	1.13 (1.02, 1.25)	0.02	0.07
105	3.4 (−2.2, 9.0)	0.23	0.49	1.07 (1.03, 1.12)	<0.001	0.01	1.01 (0.93, 1.08)	0.89	0.92
110	2.9 (−2.3, 8.0)	0.27	0.55	1.01 (0.98, 1.04)	0.57	0.70	1.03 (0.95, 1.10)	0.48	0.65
114	−3.4 (−10.0, 3.3)	0.32	0.55	1.06 (1.02, 1.11)	0.01	0.02	1.10 (1.01, 1.21)	0.03	0.10
118	−3.1 (−8.2, 2.0)	0.23	0.49	1.11 (1.07, 1.15)	<0.001	<0.001	1.08 (1.01, 1.16)	0.02	0.07
123	−7.2 (−13.0, −1.1)	0.02	0.13	1.00 (0.96, 1.04)	0.96	0.98	1.04 (0.96, 1.13)	0.34	0.53
126	2.7 (−9.3, 15.0)	0.66	0.85	1.06 (0.98, 1.15)	0.16	0.27	1.29 (1.10, 1.15)	0.001	0.01
128	−2.9 (−8.4, 2.5)	0.29	0.55	1.01 (0.98, 1.05)	0.50	0.64	1.06 (0.98, 1.14)	0.12	0.27
138	−0.2 (−4.9, 4.5)	0.94	0.95	0.95 (0.92, 0.98)	0.001	0.003	0.95 (0.90, 1.01)	0.10	0.25
146	1.9 (−3.8, 7.5)	0.52	0.72	0.94 (0.91, 0.97)	<0.001	0.002	0.98 (0.91, 1.04)	0.47	0.65
149	5.2 (0.1, 10.0)	0.05	0.21	0.93 (0.90, 0.96)	<0.001	<0.001	0.89 (0.83, 0.95)	<0.001	0.01
151	−4.0 (−10.0, 2.5)	0.23	0.49	1.05 (1.00, 1.10)	0.04	0.09	1.15 (1.06, 1.26)	0.001	0.01
153	−5.4 (−13.0, 1.8)	0.14	0.42	1.03 (0.97, 1.10)	0.33	0.48	1.07 (0.98, 1.18)	0.14	0.27
156	−0.3 (−6.2, 5.6)	0.92	0.95	1.00 (0.95, 1.05)	0.98	0.98	0.99 (0.91, 1.07)	0.75	0.84
157	−2.7 (−8.5, 3.2)	0.37	0.60	0.95 (0.91, 1.00)	0.04	0.09	1.02 (0.95, 1.10)	0.52	0.67
167	−6.6 (−12.0, −0.8)	0.03	0.14	0.97 (0.93, 1.01)	0.17	0.28	1.02 (0.94, 1.11)	0.58	0.72
169	−0.8 (−7.2, 5.7)	0.82	0.95	1.00 (0.95, 1.05)	0.96	0.98	0.95 (0.87, 1.04)	0.27	0.45
170	−5.1 (−11.0, 0.5)	0.07	0.26	1.01 (0.98, 1.05)	0.47	0.64	1.10 (1.03, 1.19)	0.01	0.04
180	4.8 (−0.3, 9.8)	0.06	0.25	1.07 (1.03, 1.10)	<0.001	0.001	1.06 (0.98, 1.14)	0.13	0.27
Immunotoxic PCBs	0.1 (−3.2, 3.4)	0.93	0.95	1.00 (0.98, 1.02)	0.96	0.98	1.00 (0.96, 1.05)	0.99	0.99
Pseudo-oestrogen PCBs	−0.9 (−3.6, 1.8)	0.50	0.72	0.98 (0.96, 1.00)	0.09	0.17	0.98 (0.94, 1.02)	0.27	0.45
Highly chlorinated anti-oestrogenic PCBs	−0.1 (−3.3, 3.1)	0.95	0.95	1.05 (1.03, 1.07)	<0.001	<0.001	1.08 (1.03, 1.12)	<0.001	0.01
Phenobarbital inducer PCBs	−0.8 (−3.2, 1.7)	0.54	0.72	1.02 (1.00, 1.03)	0.03	0.07	1.03 (1.00, 1.07)	0.03	0.11
Dioxin-like PCBs	−1.6 (−2.8, −0.5)	0.01	0.09	1.02 (1.01, 1.03)	<0.001	<0.001	1.02 (1.01, 1.04)	0.004	0.03
Ratio of pseudo-oestrogen to anti-oestrogenic PCBs	2.5 (−4.4, 9.4)	0.37	0.70	1.00 (0.95, 1.05)	0.96	0.98	0.91 (0.83, 1.00)	0.05	0.14
Total PCBs	−0.6 (−1.2, 0.1)	0.09	0.30	1.00 (1.00, 1.01)	0.08	0.16	1.01 (1.00, 1.02)	0.01	0.07
Total PCBs quartile									
1	Reference			Reference			Reference		
2	−17.0 (−31.0, −2.4)	0.02	0.13	0.82 (0.73, 0.91)	<0.001	0.002	0.96 (0.77, 1.20)	0.73	0.84
3	−10.0 (−25.0, 4.9)	0.19	0.49	0.88 (0.77, 1.01)	0.06	0.12	0.97 (0.78, 1.21)	0.80	0.86
4	−17.0 (−29.0, −5.0)	0.01	0.09	0.80 (0.71, 0.90)	<0.001	0.001	0.92 (0.77, 1.10)	0.37	0.54

^1^ A robust linear mixed regression model was fitted for sperm concentration and a Poisson mixed regression model was fitted for total motility and cells with normal morphology outcomes. Β: regression model’s coefficients. CI: confidence interval, IRR: incidence rate ratio. For sperm concentration, positive and negative coefficients are indicative of improvement and decline, while IRRs values greater or lower than 1 are indicative of improvement and decline in sperm motility or morphology, respectively. *^2^* False discovery rate correction for multiple testing. A *q*-value less than 0.05 suggests that the result is indicative of a true association.

**Table 4:** Results of season-adjusted mixed model of sperm PCB congeners, functional PCB groups, and total PCBs on sperm concentration, total motility, and cells with normal morphology. Statistically significant detrimental effects of PCB congeners on sperm quality parameters are highlighted in red, favorable effects in green.

PCB	Sperm Concentration			Total Motility			Cells with Normal Morphology		
	$\hat{\beta }$ ^1^ (95% CI)	*p*-Value	*q*-Value *^2^*	IRR ^1^ (95% CI)	*p*-Value	*q*-Value ^2^	IRR ^1^ (95% CI)	*p*-Value	*q*-Value *^2^*
18	5.6 (0.3, 11.0)	0.04	0.70	0.98 (0.96, 1.01)	0.19	0.26	0.95 (0.89, 1.01)	0.08	0.12
28	−0.7 (−5.5, 4.1)	0.78	0.86	1.04 (1.01, 1.07)	0.02	0.04	1.01 (0.95, 1.08)	0.74	0.78
31	−3.6 (−14.0, 6.2)	0.47	0.70	0.85 (0.80, 0.92)	<0.001	<0.001	0.79 (0.69, 0.90)	<0.001	0.01
81	4.6 (−5.5, 15.0)	0.37	0.70	0.93 (0.88, 1.00)	0.04	0.07	0.82 (0.72, 0.94)	0.004	0.01
95	−0.4 (−5.4, 4.7)	0.89	0.91	1.00 (0.96, 1.03)	0.80	0.80	1.01 (0.95, 1.08)	0.74	0.78
99	−0.4 (−7.7, 6.9)	0.91	0.91	1.04 (1.00, 1.09)	0.05	0.08	1.11 (1.02, 1.22)	0.02	0.04
114	1.2 (−2.7, 5.1)	0.55	0.73	0.96 (0.94, 0.98)	<0.001	0.002	0.93 (0.89, 0.98)	0.003	0.01
118	14.0 (−9.6, 38.0)	0.24	0.70	1.04 (0.89, 1.20)	0.65	0.72	1.16 (0.86, 1.58)	0.34	0.41
123	−1.2 (−5.4, 3.0)	0.59	0.73	0.99 (0.97, 1.02)	0.64	0.72	1.01 (0.97, 1.07)	0.57	0.67
151	−2.9 (−8.8, 2.9)	0.33	0.70	0.97 (0.93, 1.01)	0.18	0.25	0.99 (0.91, 1.07)	0.82	0.82
153	3.3 (−1.6, 8.3)	0.19	0.70	0.97 (0.94, 1.00)	0.03	0.06	0.94 (0.88, 1.01)	0.09	0.12
156	1.3 (−4.0, 6.6)	0.62	0.73	0.98 (0.95, 1.02)	0.33	0.40	0.92 (0.86, 0.99)	0.02	0.05
157	2.7 (−4.1, 9.5)	0.44	0.70	1.09 (1.04, 1.14)	<0.001	<0.001	1.11 (1.02, 1.21)	0.02	0.05
Immunotoxic PCBs	2.8 (−1.1, 6.6)	0.16	0.70	0.97 (0.95, 1.00)	0.02	0.04	0.95 (0.90, 1.00)	0.07	0.12
Pseudo-oestrogen PCBs	1.1 (−1.6, 3.9)	0.41	0.70	1.00 (0.98, 1.01)	0.75	0.79	0.98 (0.94, 1.02)	0.24	0.32
Phenobarbital-inducer PCBs	2.8 (−1.1, 6.6)	0.16	0.70	0.97 (0.95, 1.00)	0.02	0.04	0.95 (0.90, 1.00)	0.07	0.12
Dioxin-like PCBs	1.5 (−0.8, 3.7)	0.21	0.70	0.99 (0.97, 1.00)	0.04	0.07	0.96 (0.93, 0.99)	0.01	0.04
Total PCBs	0.6 (−0.8, 1.9)	0.41	0.70	0.98 (0.97, 0.99)	<0.001	<0.001	0.97 (0.95, 0.99)	0.003	0.01
Total PCBs quartile									
1	Reference			Reference			Reference		
2	3.3 (−9.1, 16.0)	0.60	0.73	0.83 (0.77, 0.89)	<0.001	<0.001	0.74 0.63, 0.86	<0.001	0.001
3	7.6 (−6.3, 21.0)	0.28	0.70	0.88 (0.81, 0.94)	<0.001	0.002	0.81 0.69, 0.94	0.01	0.02
4	7.6 (−6.3, 22)	0.28	0.70	0.81 (0.75, 0.88)	<0.001	<0.001	0.70 0.58, 0.83	<0.001	0.001

^1^ A robust linear mixed regression model was fitted for sperm concentration and a Poisson mixed regression model was fitted for total motility and cells with normal morphology outcomes. Β: regression model’s coefficients. CI: confidence interval, IRR: incidence rate ratio. For sperm concentration, positive and negative coefficients are indicative of improvement and decline, while IRRs values greater or lower than 1 are indicative of improvement and decline in sperm motility or normal morphology, respectively. *^2^* False discovery rate correction for multiple testing. A q-value less than 0.05 suggests that result is indicative of a true association.

## Data Availability

The data sets generated and/or analysed during the current study are not publicly available due to the sensitivity of data but are available from the corresponding authors on reasonable request (elisabetta.ceretti1@unibs.it, l.montano@aslsalerno.it).

## Supplementary Materials

The following supporting information can be downloaded at: https://www.mdpi.com/article/10.3390/toxics12010006/s1, Table S1: Spearman correlation coefficients between baseline serum and semen levels of PCB congeners; Table S2: Results of season-adjusted mixed model of serum PCB congeners, functional PCB groups and total PCBs on progressive motility. Statistically significant detrimental effects of PCB congeners on progressive motility are highlighted in red, favorable effects in green; Figure S1: Scatter plot between serum PCBs concentration and sperm concentration (106/ml): total PCBs (panel A), immunotoxic PCBs (panel B), pseudo-oestrogen PCBs (panel C), highly chlorinated anti oestrogenic PCBs (panel D), phenobarbital inducer PCBs (panel E), dioxin-like PCBs (panel F); Figure S2: Scatter plot between serum PCBs concentration and total sperm motility (%): total PCBs (panel A), immunotoxic PCBs (panel B), pseudo-oestrogen PCBs (panel C), highly chlorinated anti oestrogenic PCBs (panel D), phenobarbital inducer PCBs (panel E), dioxin-like PCBs (panel F).; Figure S3: Scatter plot between serum PCBs concentration and sperm cells with normal morphology (%): total PCBs (panel A), immunotoxic PCBs (panel B), pseudo-oestrogen PCBs (panel C), highly chlorinated anti oestrogenic PCBs (panel D), phenobarbital inducer PCBs (panel E), dioxin-like PCBs (panel F); Figure S4: Scatter plot between sperm PCBs concentration and sperm concentration (106/ml): total PCBs (panel A), immunotoxic PCBs (panel B), pseudo-oestrogen PCBs (panel C), phenobarbital inducer PCBs (panel D), dioxin-like PCBs (panel E). (Highly chlorinated anti oestrogenic PCBs were undetectable in all samples); Figure S5: Scatter plot between sperm PCBs concentration and total sperm motility (%): total PCBs (panel A), immunotoxic PCBs (panel B), pseudo-oestrogen PCBs (panel C), phenobarbital inducer PCBs (panel D), dioxin-like PCBs (panel E). (Highly chlorinated anti oestrogenic PCBs were undetectable in all samples); Figure S6: Scatter plot between sperm PCBs concentration and sperm cells with normal morphology (%): total PCBs (panel A), immunotoxic PCBs (panel B), pseudo-oestrogen PCBs (panel C), phenobarbital inducer PCBs (panel D), dioxin-like PCBs (panel E). (Highly chlorinated anti oestrogenic PCBs were undetectable in all samples).

## Abbreviations

ACN	Acetonitrile
AhR	Aryl hydrocarbon receptor
ATSDR	Agency for Toxic Substances and Disease Registry
BMI	Body mass index
CE IVD system	European Certification In Vitro Diagnostic system
CI	Confidence interval
EDCs	Endocrine-disrupting chemicals
EPIC	European Prospective Investigation into Cancer and Nutrition
GC-MS/MS	Gas chromatography tandem mass spectrometry
GLMM	Generalized linear mixed model
HPLC	High-performance liquid chromatography
HP-5 MS UI	High-performance-5 mass spectrometry ultra inert
IARC	International Agency for Research on Cancer
IPAQ	International Physical Activity Questionnaire
IQR	Interquartile range
IRRs	Incidence rate ratios
METs	Metabolic Equivalents of Tasks
PCBs	Polychlorinated biphenyls
pFDR	Positive false discovery rate
POPs	Persistent organic pollutants
PREDIMED	PREvención con DIeta MEDiterránea
PTEE filter	Polytetrafluoroethylene filter
RCT	Randomized controlled trial
WHO	World Health Organization
