# Supplementary material for: Polychlorinated Biphenyls and Semen Quality in Healthy Young Men Living in a Contaminated Area
Source: Toxics. 2023 Dec 20;12(1):6. doi: 10.3390/toxics12010006 (PMC10820147; doi:10.3390/toxics12010006)
Supplement: Supplementary file 1 [file toxics-12-00006-s001.zip › toxics-2758589-SI.pdf]

Suppl. Table S1. Spearman correlation coefficients between baseline serum and semen levels of PCB congeners

PCB	Spearman correlation coefficient
18	-0.41
28	-0.04
31	0.24
81	-0.33
95	-0.20
99	-0.34
114	0.07
118	0.02
123	0.23
151	0.04
153	0.32
156	0.28
157	0.31
Immunotoxic PCBs	0.17
Pseudo-oestrogen PCBs	0.04
Phenobarbital inducer PCBs	0.25
Dioxin-like PCBs	0.30
Total PCBs	-0.13

Suppl. Table S2. Results of season-adjusted mixed model of serum PCB congeners, functional PCB groups and total PCBs on progressive motility. Statistically significant detrimental effects of PCB congeners on progressive motility are highlighted in red, favorable effects in green.

PCB	Progressive motility		
	IRR[1] (95% CI)	p-value	q-value[2]
18	0.89 (0.82, 0.96)	**0.003**	**0.01**
28	0.98 (0.92, 1.04)	0.45	0.48
31	1.05 (0.98, 1.12)	0.14	0.18
77	1.03 (0.96, 1.10)	0.40	0.45
81	1.14 (1.04, 1.24)	**0.003**	**0.01**
95	1.01 (0.97, 1.05)	0.60	0.61
99	0.93 (0.89, 0.98)	**0.01**	**0.01**
101	1.11 (1.05, 1.18)	**<0.001**	**0.003**
105	1.05 (1.00, 1.10)	0.05	0.09
110	1.05 (1.00, 1.10)	**0.01**	**0.03**
114	1.10 (1.04, 1.16)	**<0.001**	**0.004**
118	1.12 (1.07, 1.17)	**<0.001**	**<0.001**

123	1.02 (0.97, 1.08)	0.42	0.46
126	1.14 (1.03, 1.25)	**0.01**	**0.02**
128	1.04 (1.00, 1.09)	0.06	0.09
138	0.95 (0.91, 0.98)	**0.002**	**0.01**
146	0.96 (0.92, 1.00)	0.06	0.09
149	0.91 (0.87, 0.94)	**<0.001**	**<0.001**
151	1.10 (1.04, 1.16)	**<0.001**	**0.003**
153	1.04 (0.97, 1.12)	0.30	0.36
156	0.95 (0.90, 1.00)	0.07	0.10
157	0.95 (0.90, 1.01)	0.11	0.15
167	0.96 (0.91, 1.01)	0.11	0.15
169	0.97 (0.92, 1.03)	0.38	0.44
170	1.05 (1.00, 1.09)	**0.05**	0.09
180	1.08 (1.04, 1.13)	**<0.001**	**0.002**

Immunotoxic PCBs	1.00 (0.97, 1.02)	0.89	0.89
Pseudo-oestrogen PCBs	0.97 (0.95, 1.00)	**0.04**	0.08
Highly chlorinated anti-oestrogenic PCBs	1.07 (1.04, 1.10)	**<0.001**	**<0.001**
Phenobarbital inducer PCBs	1.03 (1.01, 1.05)	**0.002**	**0.01**
Dioxin-like PCBs	1.02 (1.01, 1.03)	**<0.001**	**0.003**
Ratio of pseudo-oestrogen to anti-oestrogenic PCBs	0.96 (0.90, 1.02)	0.23	0.28
Total PCBs	1.01 (1.00, 1.01)	**0.01**	**0.01**
1	Reference		
2	0.87 (0.75, 1.00)	0.06	0.09
3	0.85 (0.72, 1.01)	0.07	0.10
4	0.74 (0.64, 0.86)	**<0.001**	**<0.001**

[1] A Poisson mixed regression model was fitted for progressive motility. CI = Confidence Interval, IRR = Incidence Rate Ratio. CI = Confidence Interval, IRR = Incidence Rate Ratio

[2] False discovery rate correction for multiple testing

SUPPLEMENTARY FIGURE LEGENDS

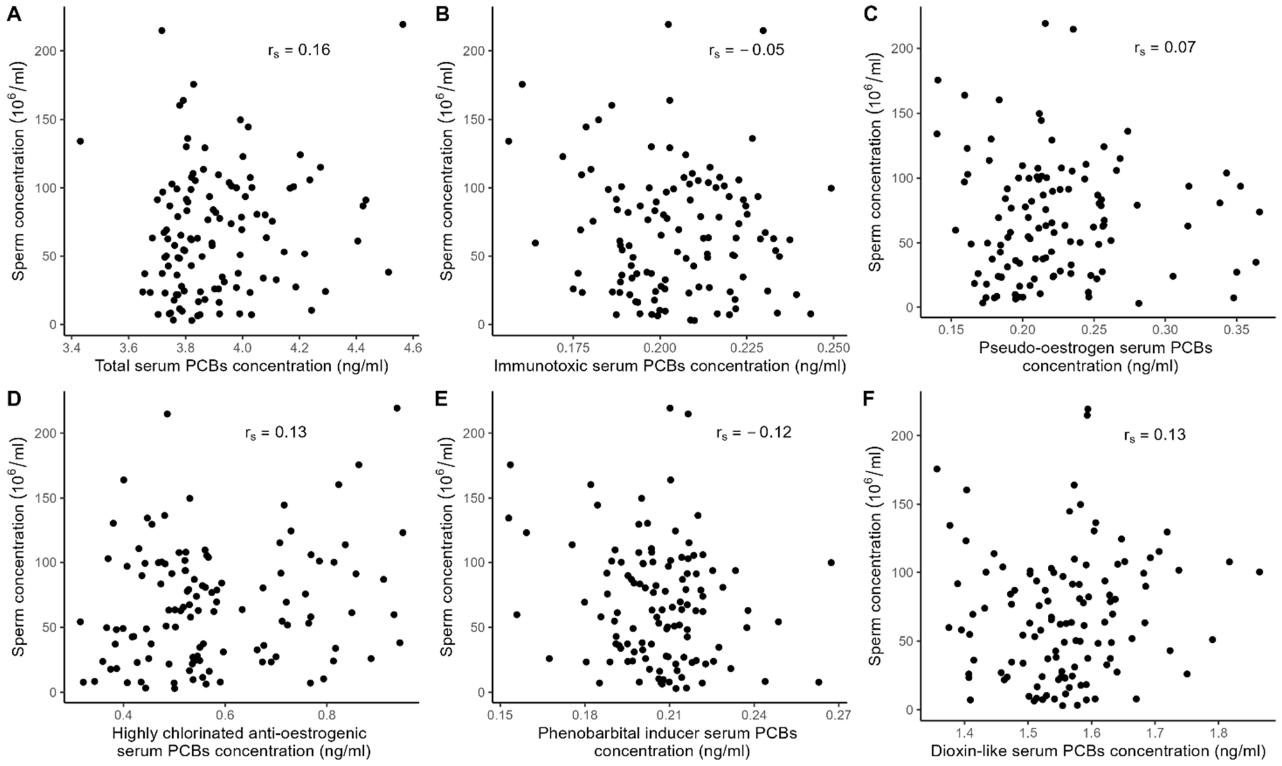

Suppl. Figure S1. Scatter plot between serum PCBs concentration and sperm concentration (10^6/ml): total PCBs (panel A), immunotoxic PCBs (panel B), pseudo-oestrogen PCBs (panel C), highly chlorinated anti oestrogenic PCBs (panel D), phenobarbital inducer PCBs (panel E), dioxin-like PCBs (panel F).

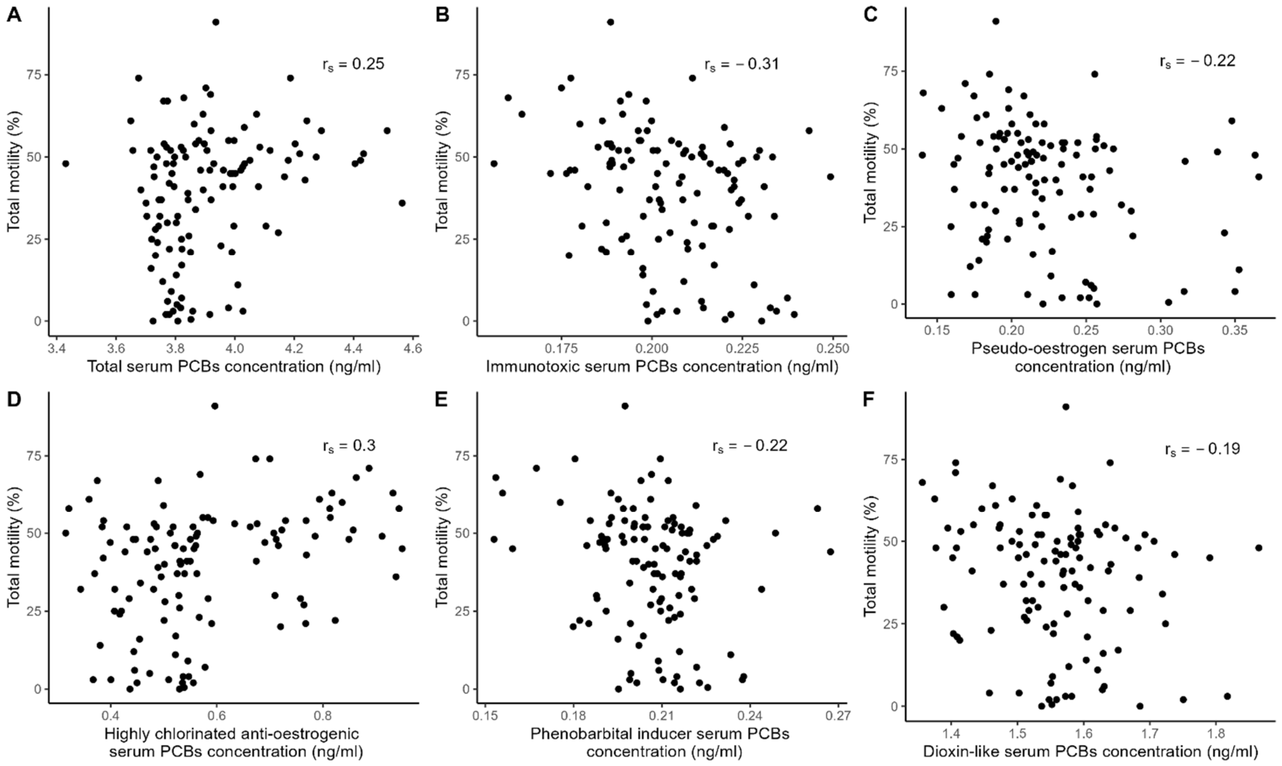

Suppl. Figure S2. Scatter plot between serum PCBs concentration and total sperm motility (%): total PCBs (panel A), immunotoxic PCBs (panel B), pseudo-oestrogen PCBs (panel C), highly chlorinated anti oestrogenic PCBs (panel D), phenobarbital inducer PCBs (panel E), dioxin-like PCBs (panel F).

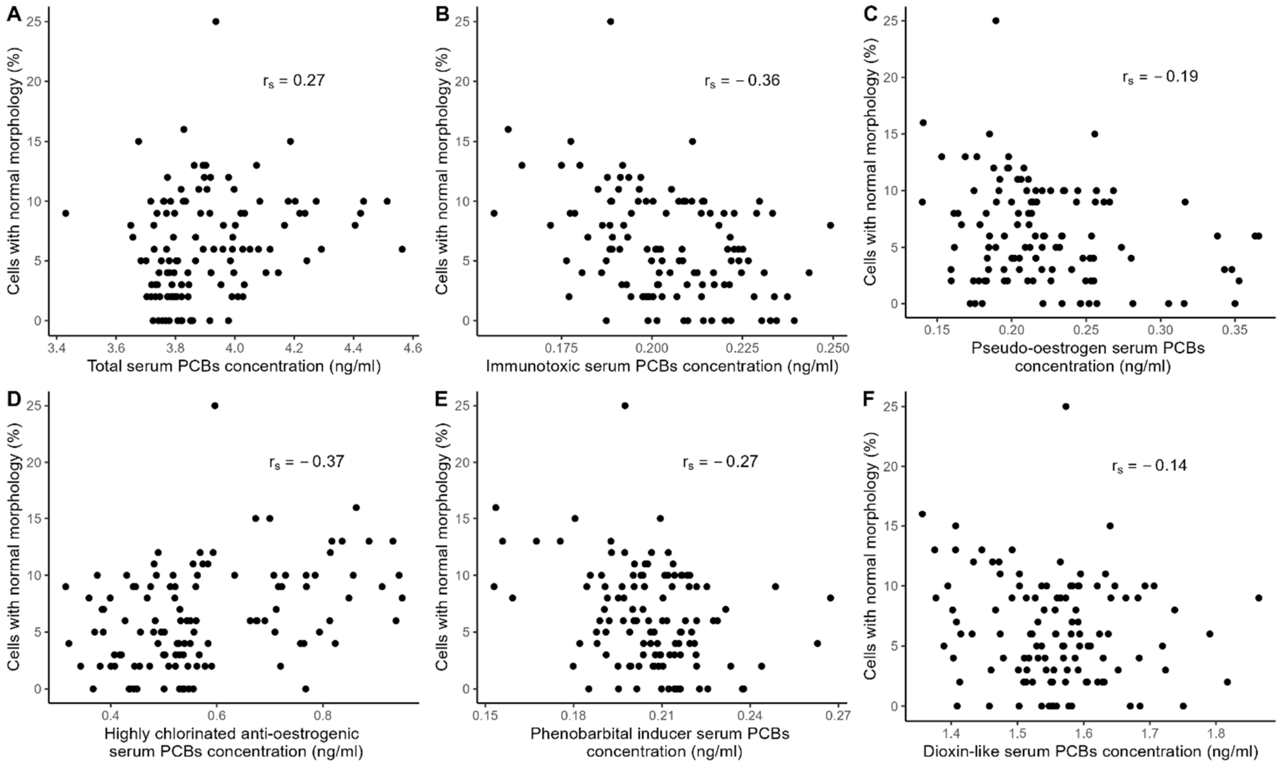

Suppl. Figure S3. Scatter plot between serum PCBs concentration and sperm cells with normal morphology (%): total PCBs (panel A), immunotoxic PCBs (panel B), pseudo-oestrogen PCBs (panel C), highly chlorinated anti oestrogenic PCBs (panel D), phenobarbital inducer PCBs (panel E), dioxin-like PCBs (panel F).

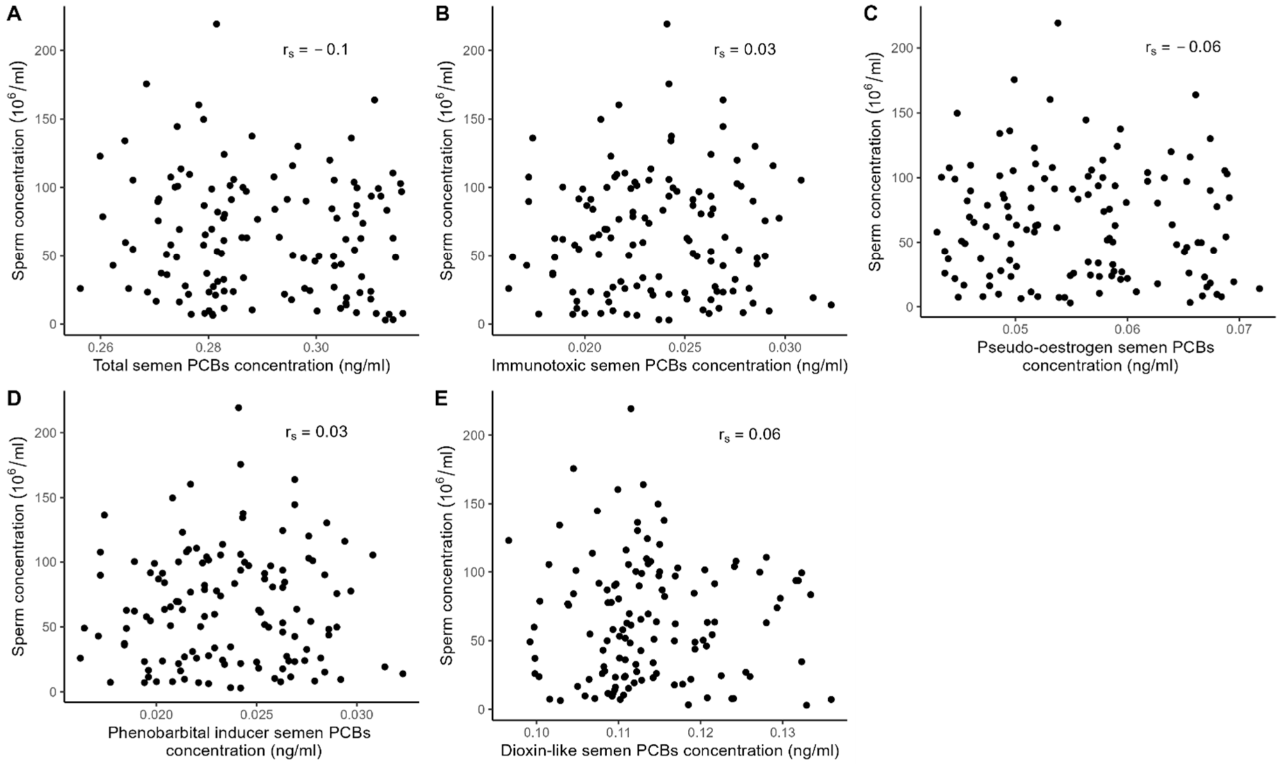

Suppl. Figure S4. Scatter plot between sperm PCBs concentration and sperm concentration (10^6/ml): total PCBs (panel A), immunotoxic PCBs (panel B), pseudo-oestrogen PCBs (panel C), phenobarbital inducer PCBs (panel D), dioxin-like PCBs (panel E). (Highly chlorinated anti oestrogenic PCBs were undetectable in all samples).

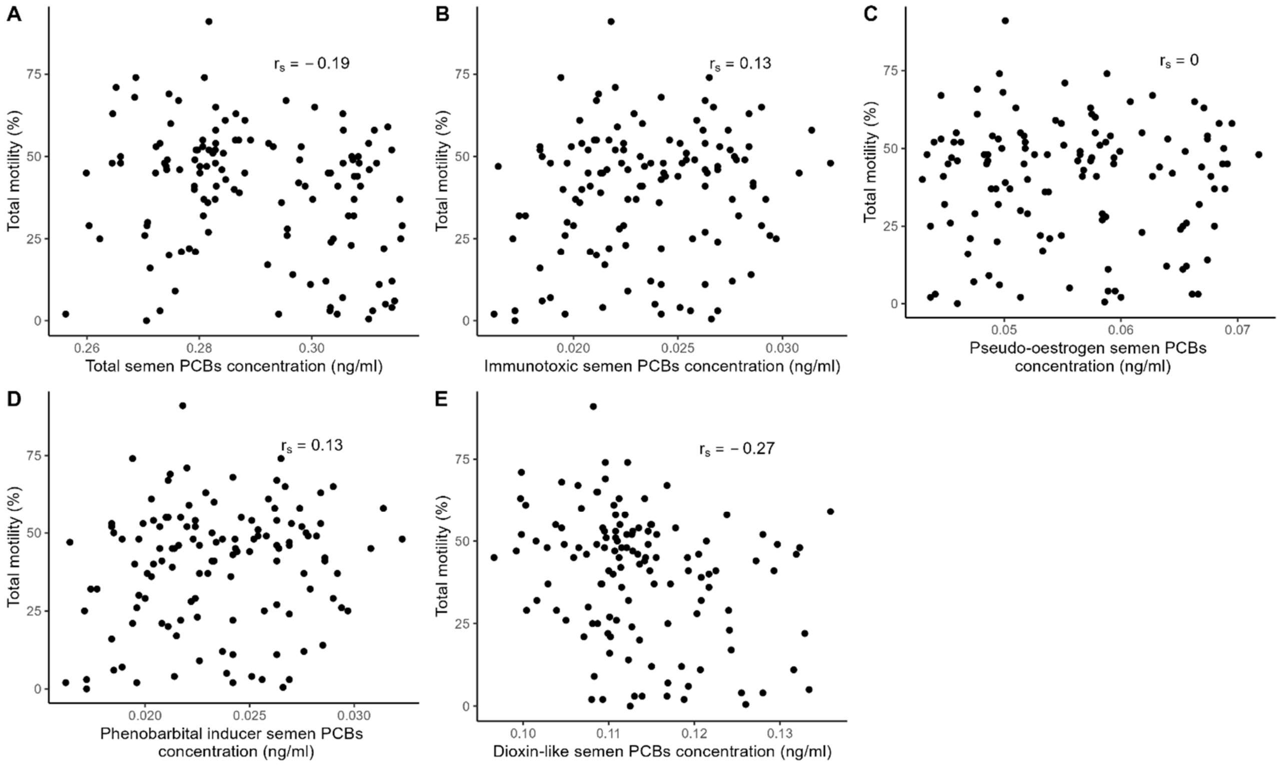

Suppl. Figure S5. Scatter plot between sperm PCBs concentration and total sperm motility (%): total PCBs (panel A), immunotoxic PCBs (panel B), pseudo-oestrogen PCBs (panel C), phenobarbital inducer PCBs (panel D), dioxin-like PCBs (panel E). (Highly chlorinated anti oestrogenic PCBs were undetectable in all samples).

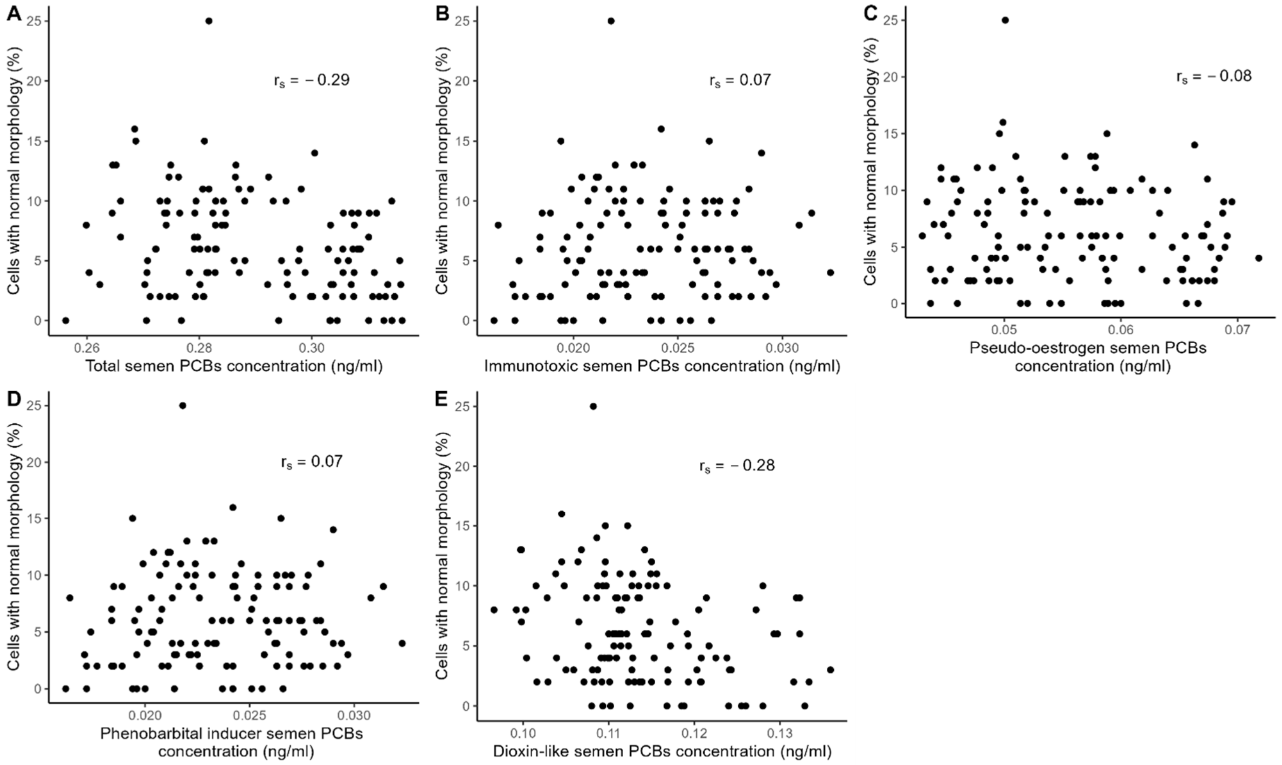

Suppl. Figure S6. Scatter plot between sperm PCBs concentration and sperm cells with normal morphology (%): total PCBs (panel A), immunotoxic PCBs (panel B), pseudo-oestrogen PCBs (panel C), phenobarbital

inducer PCBs (panel D), dioxin-like PCBs (panel E). (Highly chlorinated anti oestrogenic PCBs were undetectable in all samples).
